# Scientific and Literary Publications

**Published:** 1870-05

**Authors:** 


### Scientific and Literary Publications.

The *Technologist*, a new monthly journal, issued by the Industrial Publication Company, of New York, is especially devoted to Engineering, Manufacturing, and Building. Its publication was commenced in February, and already it has received hearty appreciation and support which it well deserves, as its articles are popular in style and soundly scientific in character.——*The Arts*, is the title of a newly started magazine, published by J. M. Hirsch & Co., in Chicago, monthly, and devoted to Science and Art. The number before us contains a lithographic portrait of the late Thomas Graham, also a biographical notice of the eminent chemist. The proceedings of the Chicago Academy of Sciences are here presented, and also news from the School of Pharmacy, recently organized in Chicago. An interesting article on the velocity of the Psychical Functions of the Brain by Prof. Hinrichs, is of interest to physicians. ——The *Atlantic Monthly*, which comes to us regularly, is cordially recommended as presenting standard essays upon the literary, political, and scientific topics of the day. Every one who has literary leisure, and is not already regularly furnished with this publication, will find it to be, in a good degree, one of his necessities.——*Littel's Living Age*, is a reprint of articles selected from English Magazines, and is published weekly.——The *American Exchange and Review*, published by Fowler & Moon, of Philadelphia, contains in the May number articles on The Organic Theory of Disease, Ethnology in its Relation to Government, Kant's Critical Philosophy, Liebeg versus Pasteurs Fermentation Theory, and other interesting contributions.

